# Supplementary material for: A mevalonate bypass system facilitates elucidation of plastid biology in malaria parasites
Source: PLoS Pathog. 2020 Feb 14;16(2):e1008316. doi: 10.1371/journal.ppat.1008316 (PMC7046295; doi:10.1371/journal.ppat.1008316)
Supplement: S1 Table — (DOCX) [file ppat.1008316.s001.docx]

| **Primer Name** | **Primer Sequence** |
| --- | --- |
| **LDH.F** | GGAGATGTAGTTTTGTTCGATATTG |
| **LDH.R** | CTTGTAAAGGGATACCACCTACAG |
| **SufB.F** | CATGTAGCTATAGTAGAAATAATAGTAAAAGATTATGG |
| **SufB.R** | GACTCTGAAATACTTAAACCACGTTGC |
| **Cox1.F** | CTTCATCTTTAAGAATAATTGCACAAGAAAATGTAAATC |
| **Cox1.R** | GGAAGCTTAGTATGGGTACATCATATGTAC |
| **pL.3LIC.NgoMIV.F** | AATTCGACAGACGCCGGCTGGTGGC |
| **pL.3LIC.NgoMIV.R** | CATGGCCACCAGCCGGCGTCTGTCG |
| **pL.5LIC.NotI.F** | GGCCGTGCCACGAGCGGCCGCTGCGC |
| **pL.5LIC.NotI.R** | TTAAGCGCAGCGGCCGCTCGTGGCAC |
| **NoPro.Adapt.F** | AGCGGCCGTGCCACGAGCGGCCGCTGCGC |
| **NoPro.Adapt.R** | TTAAGCGCAGCGGCCGCTCGTGGCACGGCC |
| **pL8.xBsaI.F** | CGGCTGGCTGGTTTATTGCTGATAAATCTGGAGCCGGTGAGCGTGGTTCTCG |
| **pL8.xBsaI.R** | ACCGCGAGAACCACGCTCACCGGCTCCAGATTTATCAGCAATAAACCAGCCAGCCGGAA |
| **LacZ BsaI** | TAAGTATATAATATTTGAGACCAAGGCTTTACACTTTATGCTTCCGGCTCGTATGTTGTGTGGAATTGTGAGCGGATAACAATTTCACACAGGAAACAGCTATGACCATGATTACGGACTCACTGGCCGTCGTTTTACAACGTCGTGACTGGGAAAACCCTGGCGTTACCCAACTTAATCGCCTTGCAGCACATCCCCCTTTCGCCAGCTGGCGTAATAGCGAAGAGGCCCGCACCGATCGCCCTTCCCAACAGTTGCGCAGTCTGAATGGCGAATGGCAGCTTGGCTGTTTTGGCGGATGAGATAAGATTTTCAGCCTGATACAGATTAAATCAGAACGCAGAAGATAAAACGAAAGGCCCAGTCGAAAGACTGGGCCTTTCGTTTTATCGGTCTCAGTTTTAGAGCTAGAA |
| **DXPR.HA1.F** | GCCACGAGCGGCCTCCATTGAAAGAAGAAAAAATAACG |
| **DXPR.HA1.R** | AAGCGCAGCGGCCCCTTCATCACCACACAATATTATAGG |
| **DXPR.HA2.F** | CGACAGACGCCGGGGTTATAGAAACCCATTTTTTATTTGA |
| **DXPR.HA2.R** | GGCCACCAGCCGGGTATATATCGGTAGCTTTATCTTTGGC |
| **DXPR.gRNA.F** | TAAGTATATAATATTTATGATGAATAAAGGTTTAGGTTTTAGAGCTAGAA |
| **DXPR.gRNA.R** | TTCTAGCTCTAAAACCTAAACCTTTATTCATCATAAATATTATATACTTA |
| **pL8HA1.R** | GCTATTTAGGTGACACTATAGAATACTCAAGC |
| **pL8HA2.F** | AATCTAGAATTCGACAGACGCCGG |
| **5'.F** | CTATTAATGATTTAGTAATAAATAATACATCAAAATGTG |
| **3'.R** | GTATTTTATTTTTTTTTGTACTATGAAGAATTATGTTTG |
| **5'WT.R** | CCTTGAAAAGAATCAATACCAATAACTATTTTATC |
| **3'WT.F** | GGAAAATGGGTAAGAAAATAACTATAGATTCTG |
| **Myco16S.F** | GGAGCAAACAGGATTAGATACCC |
| **Myco16S.R** | CACCATCTGTCACTCTGTTAACC |
