# Supplementary figures and images for: A mevalonate bypass system facilitates elucidation of plastid biology in malaria parasites
Source: PLoS Pathog. 2020 Feb 14;16(2):e1008316. doi: 10.1371/journal.ppat.1008316 (PMC7046295; doi:10.1371/journal.ppat.1008316)

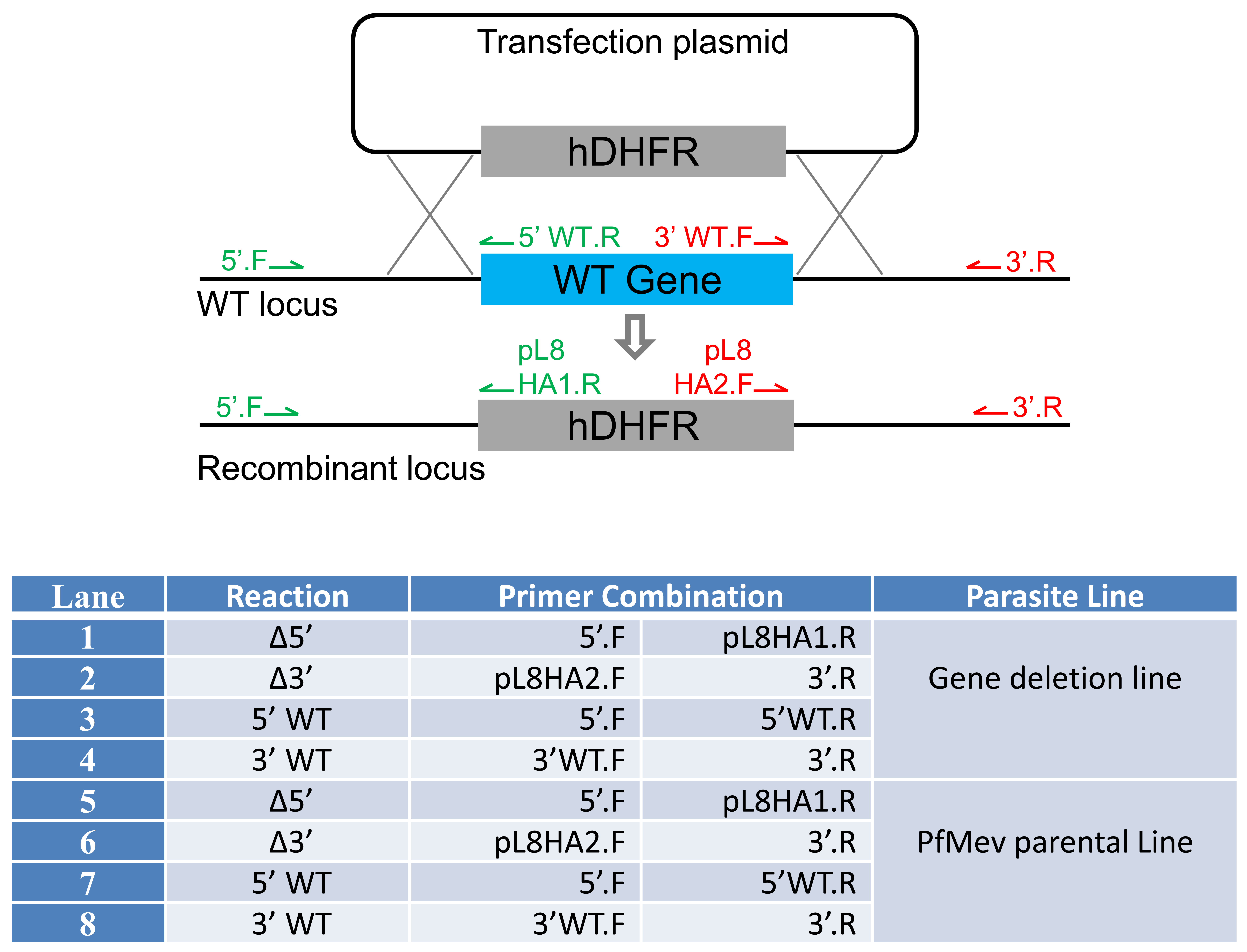

Supplement: S2 Fig — Diagram showing how the diagnostic primers listed in S1 Table were used to demonstrate integration of the hDHFR drug resistance cassette. (TIF) [file ppat.1008316.s003.tif]

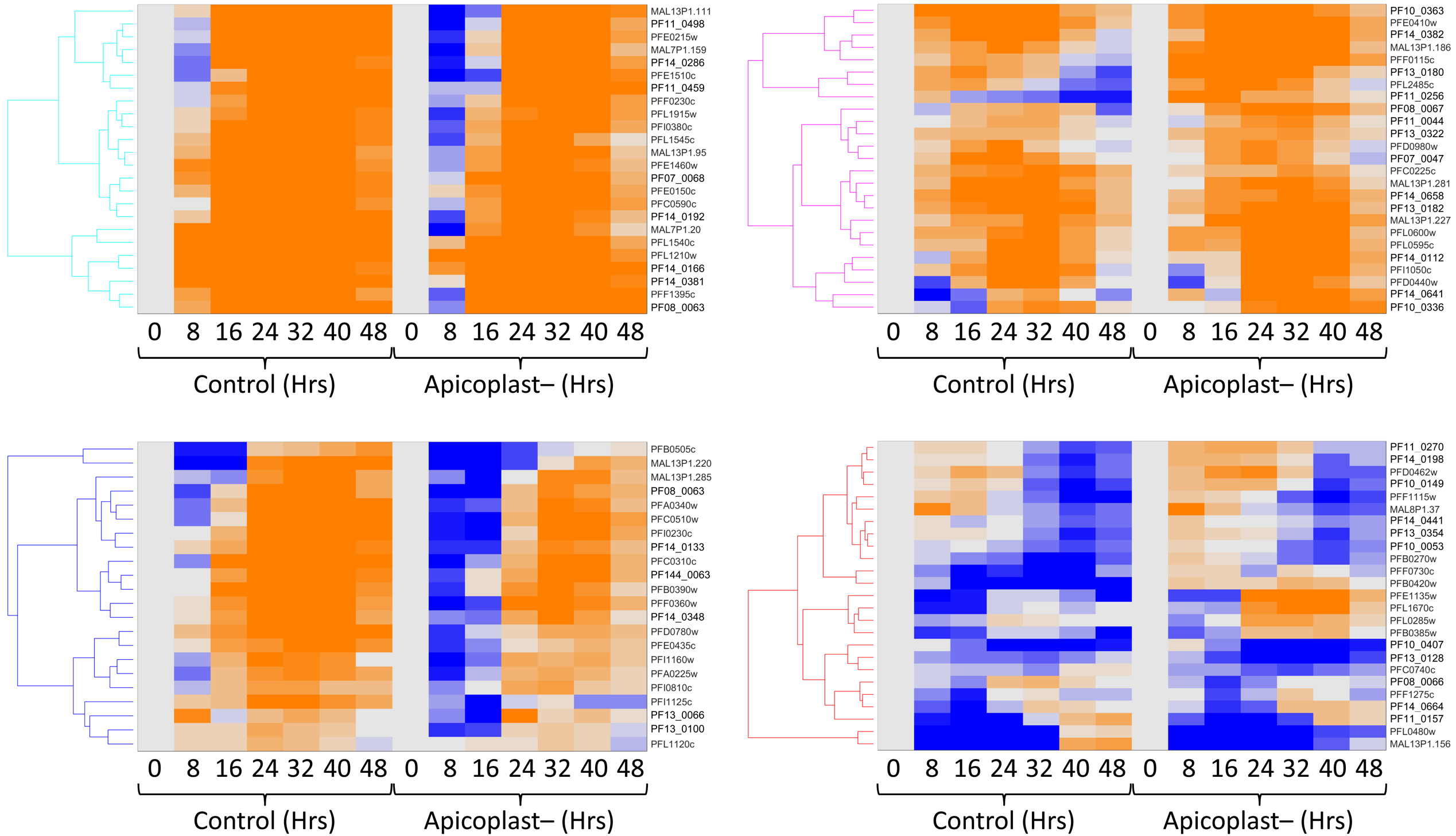

Supplement: S3 Fig — The 96 genes shown in Fig 7C can be divided into four major groups based on the hierarchical clustering of their transcriptional profiles over the 48-hour life cycle. These clusters highlight the similarities in gene expression between the control data and the data collected from apicoplast-disrupted parasites (Apicoplast-). (TIF) [file ppat.1008316.s004.tif]

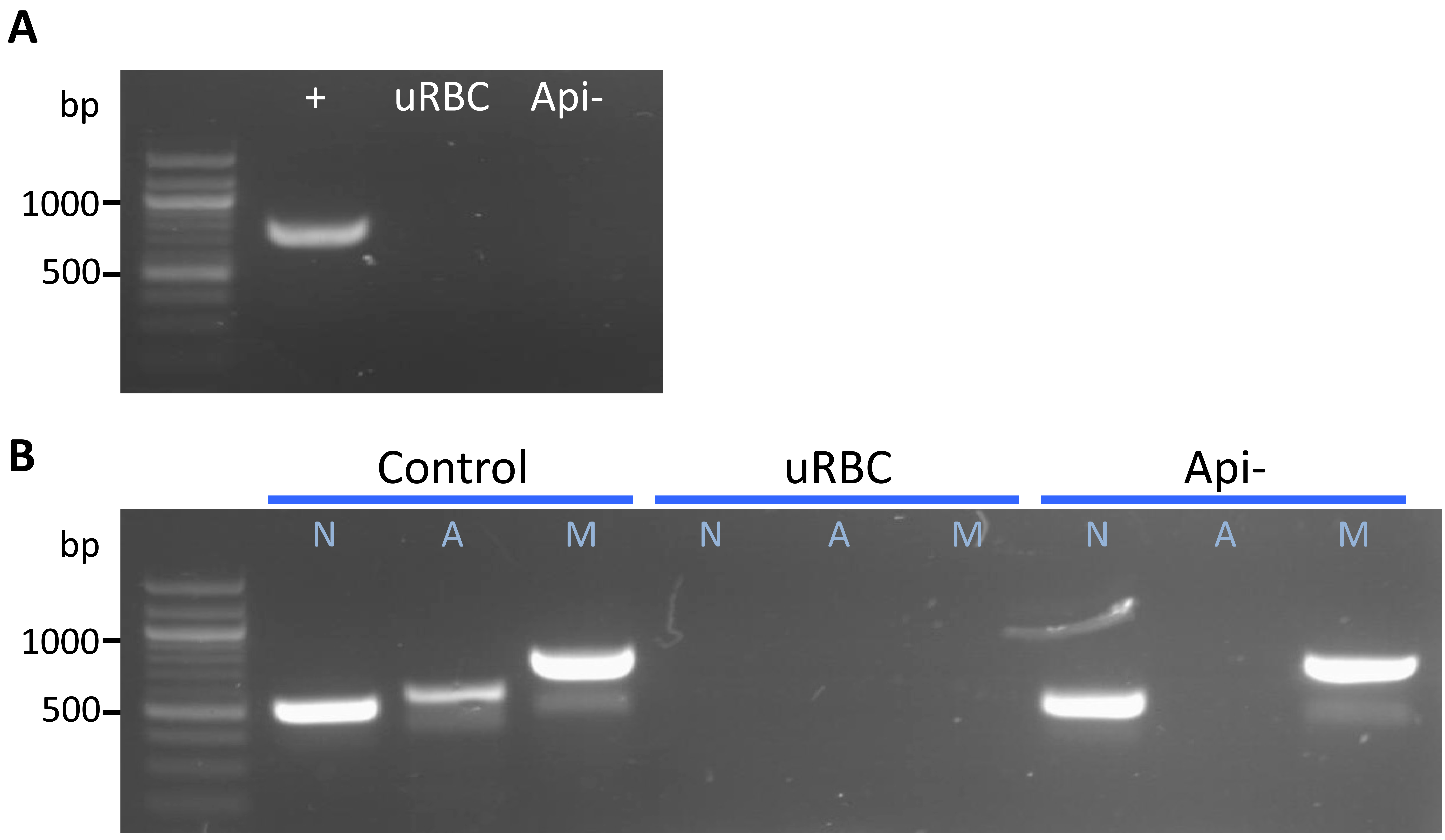

Supplement: S6 Fig — A) Lane 1 shows a PCR product generated using primers Myco16S.F and Myco16S.R with a positive control sample of Mycoplasma arginini. Lanes 2 and 3 show that the uninfected RBCs (uRBCs) and apicoplast-disrupted (Api-) parasite cultures used for transcriptomic and metabolomic experiments are not infected with mycoplasma. B) Confirmatory PCR products showing that control parasite cultures contain genes from the nuclear (N), apicoplast (A) and mitochondrial (M) genomes while the apicoplast-disrupted (Api-) parasites cultures used for transcriptomic and metabolomic experiments no longer produce a product for the apicoplast genome. Uninfected RBCs (uRBC) were used to show that PCR products are not formed in the absence of parasites. (TIF) [file ppat.1008316.s007.tif]
