# Supplementary material for: A mevalonate bypass system facilitates elucidation of plastid biology in malaria parasites
Source: PLoS Pathog. 2020 Feb 14;16(2):e1008316. doi: 10.1371/journal.ppat.1008316 (PMC7046295; doi:10.1371/journal.ppat.1008316)
Supplement: Attachment — Submitted filename: Response 15d19.docx [file ppat.1008316.s008.docx]

Reviewer #1: Swift et al have generated an alternative tool for IPP rescue by genetic engineering

parasites to utilize the lower mevalonate pathway. The major strengths of this study are the

successful metabolic engineering of Plasmodium parasites and thorough characterization of the

engineered strain. This is a tricky bit of genetic engineering in a difficult organism, including

optimization and fusing of pathway genes. An easily-addressable weakness is that the authors do

not clearly state the advantages and disadvantages of this alternative compared to the current

tool, IPP rescue, making it difficult to assess significance. The main advantage of PfMev the

authors discuss is the lower cost of the rescue compound, but this comes at the disadvantage of

additional genetic manipulation of parasites which itself is costly in time and resources. Several

of the applications (i.e. genetic transfection, compound screening, and forward genetic screens)

mentioned by the authors have been performed using IPP rescue, suggesting that IPP rescue is

sufficient to “elucidate plastid biology” for these types of experiments and not “prohibitive.”

Unfortunately, the previous literature is not sufficiently discussed or cited in the manuscript and

therefore do not allow for a fair evaluation of how PfMev improves upon the existing tool. I

agree with authors that the application w here IPP rescue is indeed prohibitive is metabolic

labeling because of lack of availability of isotope-labeled reagents. Although not necessary to

validate this tool, it would increase the significance of PfMev if more proof-of-concept for

identification of isoprenoid products could be included. In addition, this engineering is halfway

to a bypass in P. berghei for which chemical supplementation cannot easily be performed in mice;

this would indeed open up many studies of plastid biology in this in vivo model. In terms of

significance, metabolic labeling of isoprenoid products and facilitating study of apicoplast

biology in in vivo malaria models are what I find most exciting for this new tool. This work

should certainly be shared with the community, but I would recommend a more balanced

presentation about its utility compared to the existing tool of IPP rescue.

We took these comments to heart and added text to the paper, including two new paragraphs, to cover these points.

Reviewer #2: The manuscript by Swift et al. describes an elegantly conceptualized and executed

body of work that is likely to provide an excellent tool to understand plastid biology in

Plasmodium falciparum. They have generated transgenic parasites, called PfMev line, expressing

four enzymes able to utilize mavelonate to synthesize isopentenyl pyrophosphate (IPP). These

enzymes were judiciously chosen from three different organisms and were physically fused to

generate an artificial multifunctional enzyme complex, which then was expressed through a p15a

origin of replication containing vector integrated at a nonessential chromosomal locus. These

transgenic parasites were shown to become independent of apicoplast localized IPP synthesis as

long as mavelonate was provided in the medium. These parasites could be made to lose their

apicoplast when treated with azithromycin. The transcriptomic and metabolomic analysis of these

apicoplast-minus parasites revealed little alterations: transcription of genes encoding apicoplast-

targeted proteins was unaltered as were most of the metabolites. They were also able to ablate an

essential gene involved in apicoplast-localized IPP synthesis in PfMev parasites. The manuscript

is well written, experiments provide clear results, and the findings have far-reaching implications.

There are a few minor points that I would suggest to improve the manuscript:

1. Authors do not provide rationale as to of how they came up to choose the four enzymes from

3 species to generate their bypass system. It would be important to describe this in the Results

section. One has to go to the Methods section to even find out that the genes were different

species. The rationale for their choices would be instructive to readers even outside the

parasitology field.

We added a statement in the results section to indicate that this is a chimeric system and we added a paragraph to describe how these proteins were chosen in the methods section.

2. They should provide a fully annotated DNA sequence of the plasmid used to generate PfMev

parasites. There are many interesting and important aspects of this novel vector that are not

apparent from the Supplementary Fig. 1, and a deposit in GenBank is not the same as a color-

coded DNA sequence indicating various features of the plasmid included in the Supplementary

information.

We added additional detail to Supplementary Figure 1 describing some of the novel features of this plasmid, including the origin mutation and the mevalonate cassette, and submitted a fully annotated version to GenBank (MN822298). Similarly, we added a new Supplemental Figure 5 to depict plasmid pRS-LacZ and submitted an annotated version to GenBank (MN822298).

3. They do see some of the metabolites that seem to be altered in PfMev line . It would help to

provide some information about them in the Results text in addition to being listed in Fig. 8.

We added a sentence to the results section and discussed some of the specific compounds in the discussion section.

4. It would help to provide a structure of mavelonolectone in Fig. 2 and state that this is what

was used to supplement the culture medium in the Results section.

Figure 2 now shows the *RS*-mevalonolactone used through most of this work to supplement PfMev parasites and its hydrolysis to *R*- and *S*-mevalonate.

5. It would be worthwhile to point out that one possible reason for the lack of apicoplast

biosynthetic activity in apicoplast-minus parasites, in spite of having most of the apicoplast-

directed proteins in fragmented vesicles, could be the absence of a chaperone encoded by the

apicoplast genome. This could render the targeted proteins unable to form functional enzymes.

This is a good point and we added this to the discussion section in the list of reasons why proteins in these vesicles could be inactive.

Reviewer #3: This is a very well-written manuscript describing the generation, and

characterisation, of a P. falciparum parasite line that can replicate normally after the apicoplast

has been disrupted provided that mevalonate is present in the culture medium. This parasite line

can synthesise isoprenoid precursors via an exogenous, mevalonate-dependent pathway,

bypassing the parasite’s endogenous methylerythritol phosphate pathway. In characterising the

parasite line, the authors were able to delete a normally-essential enzyme (the target of

fosmidomycin). The authors also carried out metabolomic and transcriptomic studies to

investigate the consequences for the parasite of apicoplast disruption, generating valuable new

information. I believe the manuscript will be of high interest to the readership of PLOS

Pathogens and the parasite line the authors have generated will be in demand by a number of

malaria research labs around the world. I do not have any major criticism of the work but have

identified a number of minor issues which I hope the authors will address. These are listed in

order of appearance below.

We thank the reviewer for their comments and corrections below, all of which have improved the paper.

**Part II – Major Issues: Key Experiments Required for Acceptance**

Reviewer #3: No new experiments are required, but I recommend that some of the experiments

are repeated once more to generate averages from three independent experiments (see details

below).

As noted in more detail below, we conducted additional replicate experiments and all growth curves are now presented with these new data included.

**Part III – Minor Issues: Editorial and Data Presentation Modifications**

Reviewer #1: In Introduction, establishing what is possible with IPP rescue prior to this

manuscript, key citations are missing:

Use of IPP for drug screens: numerous, including Bowman et al. Anti-apicoplast and

gametocytocidal screening to identify the mechanisms of action of compounds within the

Malaria Box, Uddin et al. Validation of Putative Apicoplast-Targeting Drugs Using a Chemical

Supplementation Assay in Cultured Human Malaria Parasites, Gisselberg et al. Specific Inhibition

of the Bifunctional Farnesyl/Geranylgeranyl Diphosphate Synthase in Malaria Parasites via a

New Small-Molecule Binding Site

Use of IPP for making gene knockouts and grow otherwise non-viable transfectants: Florentin et

al. The Clp proteolytic system acts as an essential nexus for Plasmodium plastid biogenesis

(https://www.biorxiv.org/content/10.1101/718452v1.full), Gisselberg et al. The suf iron-sulfur

cluster synthesis pathway is required for apicoplast maintenance in malaria parasites

Use of IPP for forward genetic screens: Tang et al. A mutagenesis screen for essential plastid

biogenesis genes in human malaria parasites (https://doi.org/10.1371/journal.pbio.3000136)

We added a new paragraph to the introduction covering these points and including these and other references.

In Results, Design and validation of a mevalonate dependent isoprenoid synthesis pathway, the

authors mention the need for IPP isomerase (idi) for proper balancing of IPP/DMAPP levels in E.

coli. However, there is a parasite-encoded IPP isomerase annotated and no additional isomerase

is required for IPP rescue. It seems the idi may be superfluous in the parasite. Was this tested?

This was not tested. There is a publication identifying a possible IPP isomerase in malaria parasites, but this enzyme has not been studied. This is one reason why we included the characterized *E. coli* Idi enzyme in the bypass system. The other reason is that expression of MVA pathway genes kills *E. coli* unless the *E. coli idi* gene is also overexpressed. This is because the MVA pathway only produced IPP and native levels of *E. coli* Idi are not sufficient to maintain an appropriate IPP/DMAPP balance. Thus, even if there is an IPP isomerase in malaria parasites, expression of MVA pathway enzymes could overwhelm this enzyme and kill the parasites. We modified the results section to make this more clear.

In Results, Transcriptomic analysis of apicoplast disrupted parasites, the continued expression

and detection of nuclear-encoded apicoplast proteins upon loss of the apicoplast under IPP

rescue has already been demonstrated in previous papers. These should be cited, since the

literature already indicated that expression of nuclear-encoded apicoplast proteins is not affected

by apicoplast loss. The advance of this study is to show evidence for expression of many nuclear-

encoded apicoplast genes using transcriptomic analysis.

We added a sentence to this effect in the discussion section and included several references.

Was there any attempt to reconstitute the upper MVA pathway (so no chemical supplementation

is required)? Please discuss in context of potential for use in P berghei where chemical

supplementation is difficult or not possible. This would increase the significance of this work.

We added a new paragraph to discuss this topic, including the possibility and challenges of introducing this pathway in *P. berghei*.

To show utility of strain for identification of isoprenoid products, show evidence of labeled final

products, for example the volatile terpenoid compounds described in Kelly et al. This would be

very exciting and increase the significance of this work.

This is tempting to do. We showed a few labeled compounds in this work including the immediate products of the pathway, IPP/DMAPP as well as the downstream product FPP. We think that going further into the collection of final products produced by the pathway represents a new direction and is outside the scope of this report which describes the bypass system and provides examples with what can be done with it.

Fig 5. Please show the mass spectrum of the products compared to chemical standards to validate

the peaks are correctly identified.

Mass spectra of the chemical standard are now shown in Figure S4.

Reviewer #3: Minor issues.

1) In the “author summary”, IPP is referred to as “relatively unstable”. This term is too vague.

Relative to what?

Parasite culture typically involves daily feeding, so “relatively unstable” could be thought of as relative to 24hrs. We clarified this point in the author summary by stating that the half life is less than 5 hours.

2) In several places the authors refer to “as little as 10 µM”. I would suggest that the authors

refrain from using terms such as this, or explain why they consider 10 µM to be “little”. I assume

they are comparing it to how much IPP has been used in the past to allow disruption of the

apicoplast, but this is not clear (and the comparison unnecessary).

We removed this comparison, as suggested. Instead we state that parasites grow when supplemented with “at least 10 µM mevalonate.”

3) The last paragraph of the Discussion is a bit repetitive of other parts of the manuscript and can

be shortened.

The last paragraph was shortened considerably.

4) In the legend of Figure 2, the authors should clarify that the following sentence refers to

parasite treated with fosmidomycin: “PfMev parasites not supplemented with mevalonate failed

to grow”.

We modified this sentence to read “PfMev parasites treated with fosmidomycin and not supplemented with mevalonate failed to grow.”

5) The data shown in Figures 2 and 4 are from two independent experiments and the error bars

shown are SEM. SEM should not be used if the experiment has only been carried out twice. I

would encourage the authors to repeat these experiments a third time.

We conducted two additional replicates for each experiment. Figures 2 and 4 now show results from four independent experiments each conducted in quadruplicate (16 measurements per data point).

6) Legends of Figures 3, 4 and 6. The following sentence should be modified: “Images are 10

microns long by 10 microns wide”. The actual images are much bigger than that (what the authors

mean is that the size of the image can be used to represent 10 microns).

We modified the legends to state that the “images depict fields that are 10 microns long by 10 microns wide.”

7) Legend of Figure 5. To be consistent, the authors should avoid using [12C]-mevalonate and

refer to it as unlabeled mevalonate.

We made this change in the figure and the figure legend.

8) Figure 6. There is no indication of the number of independent experiments that were carried

out to generate the data shown in part D. The legend also does not indicate what the error bars

represent.

We corrected the legend text and added a replicate to this experiment. There are now three biological replicates of quadruplicate experiments (12 measurements total for each data point).

9) Figure 8 legend. The word “the” needs to be inserted in the following sentence: “The

hierarchal clustering method is THE same as described…”

We made this correction.

10) The references needs to be carefully checked to italicise species names. Reference 32 has

errors within the title. Reference 47 has a minor error next to the citation details.

We checked the references and made many corrections.

11) Figures 4, 6 and S4. Include units for 1000 and 500.

The units ‘bp’ were added to figure 4, 6 and S4.
